# Impact of postgraduate training on communication skills teaching: a controlled study

**DOI:** 10.1186/1472-6920-14-80

**Published:** 2014-04-14

**Authors:** Noelle Junod Perron, Mathieu Nendaz, Martine Louis-Simonet, Johanna Sommer, Anne Gut, Bernard Cerutti, Cees P van der Vleuten, Diana Dolmans

**Affiliations:** 1 Division of Primary Care, Department of Community Care, Primary Care and Emergency, Geneva University Hospitals, Geneva, Switzerland; 2 Unit of Development and Research in Medical Education, University of Geneva Faculty of Medicine, Geneva, Switzerland; 3 Department of Internal Medicine, Rehabilitation and Geriatric Medicine, Geneva University Hospitals, Geneva, Switzerland; 4 Geneva University Faculty of Medicine, Geneva, Switzerland; 5 Department for Educational Development and Research, Maastricht University, Maastricht, The Netherlands

**Keywords:** Communication skills, Teaching, Impact, Intervention, Direct observation, Feedback, Controlled study, Supervisors, Resident, Postgraduate

## Abstract

**Background:**

Observation of performance followed by feedback is the key to good teaching of communication skills in clinical practice. The fact that it occurs rarely is probably due to clinical supervisors’ perceived lack of competence to identify communication skills and give effective feedback. We evaluated the impact of a faculty development programme on communication skills teaching on clinical supervisors’ ability to identify residents’ good and poor communication skills and to discuss them interactively during feedback.

**Methods:**

We conducted a pre-post controlled study in which clinical supervisors took part to a faculty development program on teaching communication skills in clinical practice. Outcome measures were the number and type of residents’ communication skills identified by supervisors in three videotaped simulated resident-patient encounters and the number and type of communication skills discussed interactively with residents during three feedback sessions.

**Results:**

48 clinical supervisors (28 intervention group; 20 control group) participated. After the intervention, the number and type of communication skills identified did not differ between both groups. There was substantial heterogeneity in the number and type of communication skills identified. However, trained participants engaged in interactive discussions with residents on a significantly higher number of communication items (effect sizes 0.53 to 1.77); communication skills items discussed interactively included both structural and patient-centered elements that were considered important to be observed by expert teachers.

**Conclusions:**

The faculty development programme did not increase the number of communication skills recognised by supervisors but was effective in increasing the number of communication issues discussed interactively in feedback sessions. Further research should explore the respective impact of accurate identification of communication skills and effective teaching skills on achieving more effective communication skills teaching in clinical practice.

## Background

Communication skills are now recognized as important components of clinical skills and are trained in most medical school curricula worldwide. However, such skills are still insufficiently addressed during clerkships and postgraduate training, despite the fact that they tend to decline unless regularly recalled and practiced [1].

Observation of performance followed by feedback is considered the optimal method for teaching and assessing professionalism, interpersonal and communication skills among other competencies [2-5]. By reinforcing appropriate learning and correcting mistakes [6], this method helps learners acquire and improve clinical skills while at the same time it may indirectly improve patient care through better supervision [4]. Although students and residents value direct observation of their performance [7], observation occurs rarely [5,8-10], or “informally, without structure or dedicated time and without distinct goals” [11-13]. Untrained faculty members often feel uncomfortable observing performance and giving feedback and are unable to accurately identify skill deficiencies [14]. So, despite general agreement on the effectiveness of observation and feedback [15] and residents’ explicit demands for communication skill training [16], faculty continue to teach communication through implicit role modeling [17]. It seems that shortcomings in communication skills teaching are perpetuated by supervisors’ lack of clinical teaching skills.

Although teaching skills are considered critical to communication skills teaching [15,17-19], experts have identified two other factors that may potentially help clinical teachers identify and make use of relevant teaching moments in clinical practice [20]: 1) to agree about what is important to teach and 2) to develop teachers’ ability to recognize good, poor or mistakenly omitted skill performance. Knowing which communication skills to use or avoid in which contexts and being able to recognize skills in trainee-patient encounters seem to be two key factors for successful clinical teaching. That is why faculty development programs on communication skill training often invite teachers to participate in communication skills training to help them recognize and expand their repertoire of communication skills in various clinical situations [19,21]. The ability of supervisors to discuss the use of both poor and good communication skills in a constructive and interactive way when giving feedback to trainees is also another crucial element. However, supervisors often feel uncomfortable in providing residents negative feedback [22,23].

Several studies have reported on faculty development programs on communication skills [21,24-26], but due to poor experimental designs or lack of objective measurements, the effectiveness of programs remains undecided.

We developed an intervention aimed to train clinical supervisors to teach communication skills in practice. A first controlled study showed that the intervention was successful in that clinical supervisors used more effective feedback skills after training [27]. However, we did not assess whether it changed clinical supervisors’ ability to recognize residents’ good and poor communication skills during clinical encounters and the number and type of communication skills addressed interactively during feedback. The present study evaluated: 1) whether the intervention changed supervisors’ ability to identify the number and type of poor and good communication skills; 2) how many and which communication skills were discussed interactively by supervisors and residents during feedback sessions before and after training.

## Methods

### Design, setting and participants

We conducted a pre/post study with a control group to assess how the above described faculty development program on communication skill training impacted on supervisors’ ability to identify and discuss residents’ communication skills in two settings of the Geneva University Hospitals, Switzerland, a general internal medicine ward (inpatients) and a primary care division (outpatients). All supervisors who were available were invited to take part in the study by email and then a phone call (51 out of 65 volunteered and finally 48 participated). The intervention group consisted of 28 clinical supervisors, 16 from the inpatient setting and 12 from the outpatient setting. Twenty clinical supervisors were assigned to the control group, 12 from the inpatient setting and 8 from the outpatient setting.

The control group consisted of supervisors in the internal medicine ward who were volunteers but were not available to follow the training program and supervisors in the medical outpatient clinic of Lausanne University, Switzerland. We invited supervisors from the latter setting, because the Geneva division of primary care was too small to recruit a sufficient number of supervisors for the intervention and control groups. However, the two institutions are similar in organization and functioning and there are no reasons to expect any differences between supervisors at Geneva and Lausanne in the teaching skills addressed in the study.

### Ethics statement

The study was approved by the ethical committee of the Geneva University Hospitals. Written consent was obtained from all supervisors involved in the study.

### Intervention

**Figure 1:**
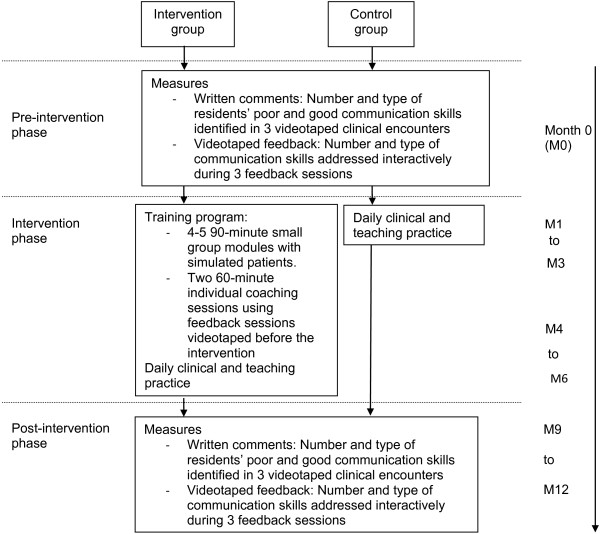
Overview of the intervention conducted and evaluation measures collected among inpatient and outpatient clinical supervisors.

The intervention consisted of a six- to nine-month faculty development program comprising four to five ninety-minute small group modules and two sixty-minute individual coaching sessions (Figure 1) [27].

#### Small group training modules

In the modules, participants were trained to play alternatively the role of a resident, a supervisor or an observer. The resident role involved interacting with a patient in a 2-3 minute encounter; the supervisor role involved identifying and giving feedback on resident’s poor and good communication skills; the observer role involved giving feedback to their colleagues about their feedback and communication skills. All participants would play these three different roles at least once during each training session under the guidance of two facilitators. NJP, MLS and JS, all experienced teachers in communication and teaching skills training, acted alternatively as group dynamic facilitators and helped extract from the role plays and small group discussions the core teaching and communication skills to be learned. The training program for the participants in the inpatient setting focused on explaining a procedure, breaking bad news and discussing with a patient’s family; in the outpatient setting, the program focused on explaining a diagnosis, managing time during a conversation with a talkative patient, and a consultation with an angry patient. At the start of each module, the facilitators asked the participants to brainstorm about which communication skills they thought would be suitable for the scenario at hand. Participants received a checklist of the most useful communication skills, derived from the Calgary Cambridge guide, which provides recommendations for communication during each medical encounter [28,29]. They were also given a checklist of the most useful feedback skills. The content of the training program was based on participants’ needs in both communication and teaching skills. However, much emphasis was put on making both patients and residents active in the interaction (exploring patients’ perspectives/residents’ needs, checking patients/residents’ understanding) and giving a structure to the clinical or teaching encounter.

#### Individual coaching sessions

During the individual coaching sessions, participants watched a videotape (nine minutes) recorded before the intervention in which they gave feedback to a simulated resident. They were first asked to self-assess their own teaching skills and identify what skills they wanted to maintain or improve. Skills to be improved were then discussed interactively and practiced through role playing. At the end of the coaching session, the facilitator asked the participant to formulate working objectives for the future. The focus of these sessions was individualized to participant’s needs. However, the importance of stimulating self-assessment, making the resident active in the solving the problem, limiting the number of items addressed and checking their understanding were often addressed.

### Outcome measures

#### Self-reported questionnaire on sociodemographic, clinical and teaching profile and self-knowledge in communication and teaching skills

Participants were asked to complete before and after intervention a self-reported questionnaire focused on socio-demographic data, clinical and teaching experience, self-knowledge in communication and teaching skills.

#### Number and type of communication skills identified during direct observation of videotaped simulated encounters

Before and after the intervention, the participants undertook three objective structured teaching encounters (OSTEs) [30] requiring them to enter in a form any good and poor communication skills they identified in videotaped encounters of a simulated resident with a simulated patient (Figure 1). The form contained separate blank spaces for comments on the good communication skills observed, the poor communication skills observed and suggestions for improvement.

Each OSTE lasted six to seven minutes. The pre- and post-intervention OSTEs contained the same communication skills: explaining a procedure, breaking bad news and a conversation with a patient’s family in the inpatient setting; and explaining a diagnosis, time management during a consultation and dealing with an angry patient in the outpatient setting. Scenarios of the videotaped encounters were developed by NJP together with a Faculty member of each service not involved in the study and displayed a pre-defined number of specific poor and good communication skills. The poor and good communication skills demonstrated by simulated residents were chosen to reflect what was frequently observed in real practice. The videotaped encounters judged by the supervisors before and after the intervention differed with respect to the socio-demographic characteristics of the patient or resident and the type of disease, but involved the same poor and good communication skills.

The first set of outcome measures were the number and the type of poor and good communication skills that the supervisors entered in the forms. Since we were interested in assessing and weighting the type of communication skills identified by supervisors, we asked three local experts in communication skills training, who had not been involved in developing the OSTEs, to individually indicate for each scenario all the poor and good communication skills used by the simulated residents and select the six poor and good communication skills they considered most relevant on the videotaped encounters. The expert list consisted of the skills that were mentioned by at least two of the experts (Table 1). The level of agreement was high and in order to limit the number of skills to six, for some scenarios, a consensus was found through discussion among experts teachers. The reported outcome measure was then the type of communication skills observed by supervisors among those considered important by the expert teachers.

**Table 1:** The six most important communication skills for different videotaped clinical scenarios identified by three experts

**Inpatient**	**Good skills**	**Poor skills**	**Outpatient**	**Good skills**	**Poor skills**
**Explaining a procedure**	Establishing initial rapport	Not exploring patient’s perspectives	**Explaining a diagnosis**	Setting the medical agenda	Not exploring patient’s perspectives
	Using clear language	No chunking nor checking		Using clear language	No chunking or checking
		No empathy			No empathy
		Not checking patient’s understanding at the end			Not checking patient’s understanding at the end
**Breaking bad news**	Warning	No agenda setting	**Managing time**	Establishing initial rapport	Not negotiating the agenda
	Exploring patient’s perspectives	Using jargon		Medical agenda setting	Not announcing the duration of the consultation
	Empathy	Not informing appropriately		Chunking	No empathy
**Discussing with a family**	Summarizing the context	Not negotiating each other’s agenda	**Managing a difficult consultation**	Agenda setting	Not exploring patient’s perspectives
	Remaining calm	Not exploring each other’s worries		Negotiating a common solution	Not acknowledging the problem
		No empathy			No empathy
		Not negotiating a common solution with the patient			No chunking or checking

#### Number and type of communication skills discussed interactively during feedback

Observation is the first step of communication skills teaching in practice. Teaching them interactively is the second important element. Since our previous study did not allow us to analyze precisely the number and type of skills addressed in an interactive way [27], the second set of outcome measures used for this study consisted of the number and the type of poor and good communication skills discussed interactively with the residents during the three videotaped feedback sessions. Feedback sessions focused on the communication skills used by simulated residents in the following issues: explaining a procedure, breaking bad news and conducting a family discussion in the inpatient setting; explaining a diagnosis, managing time and managing a difficult consultation with an angry patient in the outpatient setting. Increasing the number of skills addressed interactively was considered more relevant than increasing the overall number of communication skills addressed, since dialogue seems crucial in order to benefit from feedback [31].

Similarly to the first set of outcome measures, we focused on the type of communication skills identified by supervisors among those considered important by the expert teachers.

### Analysis

#### Self-reported questionnaire on participants’ profile and self-knowledge

We used Chi-square tests and Wilcoxon rank sum test to analyze potential differences in sociodemographic and clinical/teaching experiences between the intervention and control groups, and Wilcoxon signed ranks test to assess differences in self-knowledge in communication and teaching skills before and after intervention.

#### Communication skills identified during direct observation of videotaped encounters

The written comments were coded using a coding list based on the Calgary-Cambridge observation guide and enriched by additional communication items documented by the supervisors (Table 2). For each videotaped encounter, a maximum of 14-19 codes was defined, and these codes could be assigned a positive (+, good or well performed communication skill) or a negative (-, poor or not well performed communication skill) value. For each code, we listed a variety of examples/formulations from the comments to enable coding even if a supervisor did not use specific communication jargon. A research assistant who was blinded to supervisors’ group (intervention or control) and phase of the study (pre-post-intervention) coded all the written comments.

**Table 2:** Coding list for the six videotaped clinical scenarios

**Code**	**Communication items**	**Inpatient**			**Outpatient**		
		**Explaining**	**Breaking bad news**	**Discussing with a family**	**Explaining a diagnosis**	**Managing time**	**Managing a difficult consultation**
1	Announcing the duration of the consultation					x	
2	Establishing initial rapport	x	x	x	x	x	x
3	Setting the medical agenda	x	x	x	x	x	x
4	Introducing each other			x			
5	Negotiating the agenda			x	x	x	x
6	Announcing		x				
7	Exploring patient’s perspectives	x	x	x	x	x	x
8	Exploring patient’s understanding of what has happened						x
9	Summarizing the context	x	x	x	x	x	x
10	Informing	x	x	x	x	x	x
11	Clarifying roles, rules			x			
12	Using clear language/no jargon	x	x	x	x		x
13	Chunking and checking (by open-ended questioning, reflecting, silence)	x	x	x	x	x	x
14	Empathy/legitimating	x	x	x	x	x	x
15	Making information circulate between participants			x			
16	Being supportive	x	x	x	x	x	x
17	Summarizing	x	x	x	x	x	x
18	Planning the future	x	x	x	x	x	x
19	Remaining calm			x			x
20	Checking patient’s understanding at the end	x	x	x	x		
21	Non verbal	x	x	x	x	x	x
22	Defining the limits			x			x
23	Negotiating a common solution with the patient			x			x
24	Maintaining the frame					x	x
25	Acknowledging the problem						x
26	Apologizing						x

#### Communication skills discussed interactively during feedback

The three feedback sessions both before and after the intervention were analysed using the coding list described above. A code of 1 was assigned to a communication item if the supervisor gave feedback in an interactive way and as 0 if the feedback was a one-way process without interactivity. Communication was considered interactive if the participant involved the resident in the feedback process by inviting responses, by listening to the resident’s reaction or by asking the resident to actively formulate a solution to a problem that was identified.

NJ and AG each coded the same 10% of the forms and videos to determine interrater reliability, which was acceptable (Kappa = 0.83). We calculated means and standard deviations (SD) to summarize the number of skills observed. For each supervisor, the number of skills identified or addressed was counted in both pre and post intervention situations, and then delta, that is the difference of these two numbers, was computed.

Analysis of variance was performed to determine any group (control or intervention) effects. Effect sizes were calculated [32]. No power calculation was made before the study to find a difference between groups. All the analyses were done using TIBCO Spotfire S + ® 8.1 for Windows, TIBCO Software Corporation, Palo Alto, CA, USA.

## Results

The intervention and control groups did not differ significantly in socio-demographic characteristics and self-perceived knowledge about communication skills: the median age of the control group and the intervention group was 32.6 years (range: 28-43) and 35.5 years (range: 29-59) respectively (p = 0.06). The percentage of women was 40 in the control group and 42 in the intervention group (p > 0.99). There were no differences in years of clinical experience (8.0 years (range 3–16) in the control group and 9.5 years (range 3–26) in the intervention group, p = 0.19).

Ratings of self-perceived knowledge about communication skills on a five-point Likert scale (1 = lowest; 5 = highest) were 2.90 (SD 0.72) in the control group and 3.07 (SD 0.72) in the intervention group (p = 0.98) before the intervention; self-perceived knowledge about teaching skills was 2.25 (SD 0.79) in the control group and 2.30 (SD 0.91) in the intervention group (p = 0.16).

### Ability to identify residents’ poor and good communication skills during direct observation

There was no difference between the intervention and the control group in their ability to identify poor and good communication skills (Table 3). The only item on which the intervention group showed a tendency towards improvement after the intervention was the ability to identify good communication skills in the consultation with a talkative patient in the outpatient setting.

**Table 3:** **Mean number of communication skills** (**CS**) **identified by supervisors during direct observation of videotaped clinical encounters**

**Ability to identify residents’ good and poor CS**		**Intervention group (Inpatient n** **=** **16; outpatient n** **=** **12)**			**Control group (Inpatient n** **=** **12; outpatient n** **=** **8)**					
		**Pre**	**Post**		**Pre**	**Post**				
		**Mean (SD)**	**Mean (SD)**	**Delta**	**Mean (SD)**	**Mean (SD)**	**Delta**	**Delta difference**	**p**	**Effect size**
INPATIENT SETTING	**To explain a procedure**									
	No. of good CS	2.38 (1.26)	2.19 (1.11)	-0.19 (1.38)	2.25 (0.97)	2.17 (1.11)	-0.08 (1.24)	-0.10	0.8380	-0.08
	No. of poor CS	4.56 (1.59)	3.94 (1.29)	-0.63 (2.28)	4.00 (1.04)	4.75 (1.60)	0.75 (1.60)	-1.38	0.0862	-0.86
	**To break bad news**									
	No. of good CS	3.25 (1.06)	4.31 (1.25)	1.06 (1.29)	4.00 (1.41)	4.67 (1.30)	0.67 (1.92)	0.40	0.5198	0.21
	No. of poor CS	4.38 (1.75)	3.75 (1.61)	-0.63 (1.89)	4.25 (1.42)	4.50 (1.93)	0.25 (1.91)	-0.88	0.2390	-0.46
	**To conduct a family discussion**									
	No. of good CS	2.94 (1.48)	2.38 (0.96)	-0.56 (1.97)	3.42 (1.44)	3.67 (1.56)	0.25 (1.96)	-0.81	0.2884	-0.41
	No. of poor CS	4.25 (1.81)	4.50 (1.79)	0.25 (2.65)	3.58 (1.00)	3.58 (1.83)	0.00 (1.91)	0.25	0.7838	0.13
OUTPATIENT SETTING	**To explain a diagnosis**									
	No. of good CS	1.33 (1.23)	3.00 (1.65)	1.67 (2.02)	2.75 (1.58)	3.14 (0.90	0.14 (1.95)	1.52	0.1263	0.78
	No. of poor CS	3.92 (2.50)	3.41 (2.27)	-0.50 (3.73)	3.50 (2.27)	1.43 (0.98)	-2.00 (3.00)	1.50	0.3787	0.50
	**To manage time**									
	No. of good CS	2.25 (1.42)	3.25 (1.48)	1.00 (1.71)	3.75 (1.67)	3.14 (2.12)	-0.86 (1.21)	1.86	0.0221	1.53
	No. of poor CS	2.33 (1.37)	3.08 (1.16)	0.75 (1.86)	1.88 (1.46)	2.14 (1.07)	0.57 (0.98)	0.18	0.8182	0.18
	**To manage a difficult relationship**									
	No. of good CS	1.50 (1.00)	2.17 (1.47)	0.67 (1.61)	1.75 (1.39)	3.14 (1.95)	1.43 (2.76)	-0.76	0.4542	-0.28
	No. of poor CS	3.92 (1.68)	4.25 (1.71)	0.33 (2.23)	3.63 (1.41)	2.43 (0.98)	-1.00 (2.16)	1.33	0.2207	0.62

If Bonferroni correction was applied, p < 0.004 (0.05/12) is significant.

On average, the supervisors from both groups identified around 30 to 60% of the six poor and good communication skills identified by the experts before and after intervention. A detailed analysis showed that the type of items identified by supervisors varied substantially independently of the communication situations, phases of the study and groups (data not reported).

### Ability to address residents’ communication skills in an interactive way

Supervisors in the intervention group addressed a higher number of communication skills in an interactive way with their residents (Table 4): this was the case especially for poor communication skills in all inpatient scenarios (effect sizes 0.80 to 1.77); outpatient supervisors in the intervention group discussed interactively a higher number of positive communication skills in the scenarios “explaining a diagnosis” and to a lesser extent “managing a difficult doctor-patient relationship” (effect sizes 0.52 to 1.77). Eight of the nine communication skills which were discussed significantly more interactively with residents after the intervention were all part of the skills considered important by the expert teachers (Table 5).

**Table 4:** **Mean number of skills** (**CS**) **addressed interactively by supervisors during feedback sessions**

**Ability to identify residents’ good and poor CS**		**Intervention group (Inpatient n** **=** **16; outpatient n** **=** **12)**			**Control group (Inpatient n** **=** **12; outpatient n** **=** **8)**					
		**Pre**	**Post**		**Pre**	**Post**				
		**Mean (SD)**	**Mean (SD)**	**Delta**	**Mean (SD)**	**Mean (SD)**	**Delta**	**Delta difference**	**p**	**Effect size**
INPATIENT SETTING	**To explain a procedure**									
	No. of good CS	0.13 (0.34)	0.69 (0.95)	0.56 (0.96)	0.00 (0.00)	0.08 (0.29)	0.08 (0.29)	0.48	0.1089	1.66
	No. of poor CS	0.81 (0.91)	3.81 (1.33)	3.00 (1.37)	0.41 (0.67)	1.50 (1.31)	1.08 (1.24)	1.92	0.0008	1.55
	**To break bad news**									
	No. of good CS	0.00 (0.00)	1.00 (1.21)	1.00 (1.21)	0.18 (0.40)	0.33 (0.49)	0.18 (0.60)	0.82	0.0497	1.36
	No. of poor CS	0.75 (0.68)	2.69 (1.35)	1.94 (1.53)	0.91 (0.94)	0.92 (1.00)	0.09 (1.04)	1.85	0.0019	1.77
	**To conduct a family discussion**									
	No. of good CS	0.07 (0.26)	0.81 (0.91)	0.73 (0.88)	0.08 (0.29)	0.17 (0.58)	0.08 (0.67)	0.65	0.0453	0.97
	No. of poor CS	1.13 (1.26)	3.19 (1.05)	2.06 (1.69)	1.00 (1.35)	1.25 (1.29)	0.25 (2.26)	1.81	0.0223	0.80
OUTPATIENT SETTING	**To explain a diagnosis**									
	No. of good CS	0.40 (0.70)	0.80 (1.14)	0.40 (0.52)	0.50 (0.53)	0.00 (0.00)	- 0.50 (0.53)	0.90	0.0023	1.68
	No. of poor CS	1.40 (1.43)	2.80 (1.14)	1.40 (1.65)	2.00 (1.20)	2.25 (1.83)	0.25 (1.58)	1.15	0.1536	0.73
	**To manage time**									
	No. of good CS	0.50 (0.67)	1.00 (0.74)	0.50 (0.80)	0.25 (0.46)	0.75 (1.16)	0.50 (1.20)	0.00	1.00	0.00
	No. of poor CS	2.33 (1.97)	2.58 (0.79)	0.25 (2.26)	1.25 (1.04)	2.13 (0.83)	0.88 (0.83)	-0.62	0.4670	-0.75
	**To manage a difficult relationship**									
	No. of good CS	0.25 (0.62)	2.25 (0.87)	2.00 (1.21)	0.38 (0.52)	1.13 (0.83)	0.75 (0.71)	1.25	0.0170	1.77
	No. of poor CS	2.33 (1.78)	1.75 (1.06)	-0.58 (1.68)	2.25 (1.75)	1.00 (0.93)	-1.25 (1.28)	0.67	0.3540	0.52

If Bonferroni correction is applied, p is significant at 0.004 (0.05/12).

**Table 5:** Type of CS addressed interactively among the items identified as important by experts

		**Intervention group**			**Control group**					
		**(Inpatient n** **=** **16; outpatient n** **=** **12)**			**(Inpatient n** **=** **12; outpatient n** **=** **8)**					
**Type of CS items**		**Pre**	**Post**		**Pre**	**Post**				
		**n (%)**	**n (%)**	**Delta** (**SD**)	**n (%)**	**n (%)**	**Delta (SD)**	**Delta difference**	**p**	**Effect size**
**Explanation inpatient**	Not setting the agenda (-)	0 (0%)	6 (38%)	0.38 (0.50)	0	0	0	0.38	0.0157	0.90
	Not exploring patients’ perspectives (-)	1 (6%)	15 (94%)	0.88 (0.34)	1 (8%)	4 (33%)	0.25 (0.45)	0.63	0.0003	1.38
	Not chunking nor checking (-)	2 (13%)	12 (75%)	0.62 (0.50)	0	2 (17%)	0.17 (0.39)	0.46	0.0142	1.77
	Not planning the follow-up (-)	5 (31%)	2 (13%)	-0.19 (0.54)	1 (8%)	4 (33%)	0.25 (0.45)	-0.44	0.0325	-0.97
	Not checking patient’s understanding at the end (-)	1 (6%)	10 (63%)	0.56 (0.51)	1 (8%)	3 (25%)	0.17 (0.58)	0.40	0.0663	0.696
**Breaking bad news inpatient**	Not setting the agenda (-)	0	5 (31%)	0.31 (0.48)	1 (8%)	0	-0.09 (0.30)	0.40	0.0207	1.34
**Family discussion inpatient**	Summarizing the context (+)	0	5 (31%)	0.31 0.48)	0	0	0	0.31	0.0331	0.80
**Difficult consultation outpatient**	No empathy (-)	1 (8%)	10 (83%)	0.75 (0.62)	2 (25%)	12 (13%)	-0.13 (0.35)	0.88	0.0021	2.48
	Negotiating a common solution (+)	2 (17%)	10 (83%)	0.67 (0.65)	2 (25%)	2 (25%)	0 (0.54)	0.67	0.0274	1.25

If Bonferroni correction is applied, p is significant at 0.00016 (0.05/308).

## Discussion

The results show that a faculty development program on communication skills teaching had no impact on clinical supervisors’ ability to recognize good or poor communication skills. The type of communication skills identified by individual participants varied considerably irrespective of the clinical scenario, intervention or control group or pre- or post-intervention phase. However, the intervention had positive effects on the number and type of communication skills discussed interactively during feedback irrespective of the situation at hand. Inpatient supervisors discussed a higher number of poor skills after the intervention.

After the intervention we expected that the numbers of good and poor communication skills identified by the intervention group would increase. The failure of this increase to materialize may be explained by several factors: a first explanation may be that participants were given blank forms in which to enter their comments. Such forms have been shown to be less effective than more specific and detailed forms in documenting strengths and weaknesses of clinical skill performance [33,34]. However, given the purpose of our study, we did not want to use checklists because it would have artificially induced a higher recognition rate of communication skills. We also wished to reproduce the type of support that supervisors were likely to provide to residents during their daily activities. Second, the time devoted to this aspect during the faculty development program was slightly shorter compared to teaching skills, since only the 4-5 small group modules addressed communication skills issues. Third, supervisors may have been more eager to learn about teaching than communication skills since their self-reported knowledge in teaching skills was lower than in communication skills. Finally, the absence of measurable improvement may be attributable to the outcome variables being not sufficiently sensitive.

We found strong heterogeneity in the type of communication skills identified by supervisors. Generally, 30 to 60 per cent of the communication skills identified as crucial by the expert panel were identified by the supervisors. These findings are consistent with previous studies showing that reliable evaluation of communication skills remains quite a challenge [35,36]. Insufficient teaching practice may again be a plausible explanation. Contrary to experts in communication skills teaching who tend to use the same theoretical framework in teaching communication skills, less experienced teachers may not have recourse to a similar framework and consequently identify skills that make sense to them in relation to the context, their perceptions, values and prior experiences. Although considerable efforts have been made to define essential elements of effective doctor-patient communication and provide a coherent framework for teaching and training [37], much remains to be clarified about which type of behavior or communication is preferable in a given clinical situation [38]. A lack of criteria leaves room for subjectivity and may explain the variety in perceptions reported by supervisors. Independent of training, communication has been described as being inherently subjective and dependent on what it means to patients and doctors in a specific context [39]. Similarly, the focus of communication skill teaching may change according to the meaning clinical supervisors give to certain communication issues in different contexts.

A way to improve participants’ ability to identify more relevant skills may be to specifically train them to rate residents’ skills using a checklist. However, giving the importance of being aware of one’s own communication patterns when teaching communication skills, training supervisors to rate their own communication skills on videotaped clinical encounters may be a more meaningful way to help them internalize the most essential skills to recognize and use in different situations. Furthermore, more training hours are needed than 8-9 hours over a period of 6 months.

Finally, the results showed improvement in feedback with the intervention group giving interactive feedback on a higher number of poor and good communication skills after the intervention and essentially on skills considered important by expert teachers and emphasized during the training. It suggests that trained supervisors selected more effectively the type of communication skills to discuss during feedback and did not avoid addressing poor performance. Effective communication skills teaching includes not only correct identification of relevant communication skills but also the ability to chose which skills to address interactively during feedback and also to give negative feedback. Negative feedback seems to stimulate deeper reflection [40]. These positive findings are of importance since feedback is more effective when it is specific, focuses on a limited number of elements and actively involve learners in the process of learning [41,42]. Small group and individual video-based coaching sessions both provide opportunities for self-reflection, practice and rehearsal and feedback [15]. However, the individual video-based sessions which took place three months later may have favored more on in-depth behavioral changes.

One limitation of the study is the limited number of participants in each setting preventing slight differences from reaching statistical significance. Another limitation is that written comments may not cover all communication skill items identified by participants. Coding, counting and listing the type of communication skills observed and documented may not be the only way to capture changes in the ability to recognize communication skills. Finally, the results were derived from simulated feedback sessions and further research will have to determine whether there was an actual improvement in the quality of communication skill teaching by participants.

## Conclusions

In conclusion, this study shows that a 8-9 hour faculty development program on communication skills teaching improved supervisors’ ability to discuss important communication issues in an interactive way but failed to increase their ability to identify a higher number of communication skills displayed by residents. More specific training of clinical supervisors in rating of residents’ communication skills together with the use of more detailed observation forms (e.g. checklists) during pre- and post evaluation phases may increase their ability to identify residents’ poor and good communication skills. Although both aspects would be ideal for teaching purposes, the limited number of observed skills reported by supervisors did not prevent an improvement in the intervention group in giving feedback to residents. Further research should explore the respective impact of accurate identification of communication skills and effective teaching skills on improving communication skills in clinical practice.

## Competing interests

The authors declare that they have no competing interests.

## Authors’ contributions

NJP, MN, CV and DD conceived and designed the study. NJP, AG, JS, MLS and MN participated to the data collection and analysis. NJP, MN and BC carried out the analysis. NJP wrote the first draft of the manuscript. All other authors critically revised the manuscript and gave approval to the final version.

## Pre-publication history

The pre-publication history for this paper can be accessed here:

http://www.biomedcentral.com/1472-6920/14/80/prepub
